# Developmental and sequenced one-to-one educational intervention (DS1-EI) for autism spectrum disorder and intellectual disability: a two-year interim report of a randomized single-blind multicenter controlled trial

**DOI:** 10.1186/s12887-020-02156-z

**Published:** 2020-05-29

**Authors:** Antoine Tanet, Annick Hubert-Barthelemy, Marie-Noëlle Clément, François Soumille, Graciela C. Crespin, Hugues Pellerin, François-André Allaert, David Cohen, Catherine Saint-Georges, the GPIS study group, Véronique Bur, Aude Brellier, Christophe Chartier, Claire Ducateau, Jean-Louis Sarradet, Danièle Scellier, Hélène Petiton, Marc Colombel, Marc Bandelier, Louisa Garnil, Emmanuel Damville, Pierre Delion, Isabelle Gylbert, Anne Juteau, Anne Vautrin, Jean-François Havreng, Elisabeth Simonet, Georges Lançon, Yves Faure, Aurélie Broche, Myriam Garing, Caroll Devaux, Sophie Michalak

**Affiliations:** 1 Institut des Systèmes Intelligents et de Robotiques, Sorbonne Université, 75005 Paris, France; 2 Département de Psychiatrie de l’Enfant et de l’Adolescent, APHP, Groupe Hospitalier Pitié-Salpêtrière et Sorbonne Université, 75013 Paris, France; 3 Association Programme de Recherche et d’Etudes sur l’Autisme, 7, square Dunois, 75013 Paris, France; 4 Hôpital de jour André Boulloche, association Cerep-Phymentin, 56 rue du Faubourg Poissonnière, 75010 Paris, France; 5 Association Régionale pour l’Intégration, 26 rue Saint Sébastien, 13006 Marseille, France; 6 CEN Biotech, Parc Mazen-Sully, Zone des biotechnologies, Impasse Françoise Dolto, 21000 Dijon, France

**Keywords:** Autism, Intellectual disability, Randomized controlled trial, Special education, Developmental intervention

## Abstract

**Background:**

Children with autism spectrum disorder (ASD) and moderate to severe intellectual disability (ID) face many challenges. There is little evidence-based research into educational settings for children with ID and ASD and in France. Little is known about how this unserved population could benefit from intervention and education. This study assessed the feasibility and efficacy of a new intervention model using an individualized educational approach.

**Methods:**

We conducted a randomized, single-blind controlled trial to assess a novel intervention: the “Developmental and Sequenced One-to-One Intervention (DS1-EI)”. In DS1-EI, trained teachers worked one-to-one with each child in a small classroom setting, offering 10 h per week of the intervention. The focus was on encouraging spontaneous communication, promoting skills through play with peers, supporting positive interactions, and developmental and sequenced learning. We enrolled 5- to 9-year-old children with ASD and ID across 11 French child care institutions for children with co-occurring ASD and ID. Participants were matched in dyads by developmental quotient and randomized to the treatment-as-usual (TAU) group or the DS1-EI group. Independent raters blindly assessed the primary variables: The Childhood Autism Rating scale (CARS) and the Psychoeducational Profile, third edition (PEP-3). The secondary variables included the Vineland Adaptive Behavior Scale II (VABS-II) and the Clinical Global Assessment Scale (CGAS). Here we perform interim analyses at 24 months.

**Results:**

At baseline, 72 participants were randomized. Nine patients (5 in the DS1-EI group and 4 in the TAU group) dropped out of the study. Using linear mixed models, both intent-to-treat (ITT) and per-protocol (PP) analyses at the 12-, 18- and 24-month outcomes showed no significant group nor group-by-time interaction effects. However, we found significant improvements in most primary and secondary variables over time in both groups.

**Conclusions:**

The study did not show that DS1-EI was superior to TAU in treating children with ASD and ID over 24 months. However, the low dropout rate shows that DS1-EI is feasible, and well accepted. As the study is still ongoing, we need to wait for data at 36 months to ensure whether DS1-EI could be recommended.

**Trial registration:**

ANSM130282B-31 (April 16, 2013) and ACTRN12616000592448. Registered 6 May 2016, retrospectively registered, http://www.anzctr.org.au/

## Background

Autism spectrum disorders (ASD) are defined as socio-communicative disorders beginning very early in life that are associated with atypical patterns of sensorimotor behavior or restrictive behaviors or interests [1]. The etiology includes both biological (e.g., genetic conditions) and environmental factors that affect neurodevelopment [2].

Despite the growing evidence regarding behavioral and neurobiological pervasive developmental trajectories in children with ASD, no agency approved biological treatment exists for ASD, and the therapeutic approach is mainly provided by clinicians focusing on the development and behavior of the child [3]. The USA National Standards Report on evidence-based practice guidelines [4] concluded that there is currently no single effective treatment for ASD and underscored the importance of a multimodal treatment approach.

The effects of several therapeutic programs have been assessed through moderate-to-high-quality studies. The most widely-recognized programs include Treatment and Education of Autistic and Communication Handicapped Children (TEACCH) [5, 6], Applied Behavioral Analysis (ABA) [7] and its derivative Pivotal Response Training (PRT) [8], the Early Start Denver Model (ESDM) [9] and the Developmental, Individual-Differences and Relationship-based (DIR) Model [10, 11]. Other alternative developmental approaches based on play therapy, such as the Son-Rise Program® [12], Exchange and Development Therapy [13], and the 3i method [14] have been investigated, but only in the context of pilot studies. Parent-mediated social communication therapy (PACT) has been found to have a positive impact at long term follow-up when the intervention is introduced early [15]. As shown in various systematic reviews and meta-analyses, the effectiveness of behavioral and educational interventions for ASD is well-documented [16, 17]. Notably, several reviews showed that early intensive intervention (EIBI) is effective for young children with ASD [18–21]. However, in a review of 34 studies, Warren (2011) [22] concluded there was insufficient evidence to determine which specific interventions are the most effective treatments for children with ASDs. A more recent meta-analysis [23] made the same conclusion when comparing three types of intervention (behavioral, social communication focused, and multimodal developmental): none showed a reduction in autism severity, and there was no significant difference among the intervention types. Another meta-analysis [24] found that music therapy appears to be the most effective tool for improving social interaction in preschool-aged children with ASD. Thus, despite the RCT evidence base to date, new studies are needed to investigate the strengths and weaknesses of each intervention, to determine which particular intervention or combination could be the most effective and to assess how interventions could be tailored to each child.

Narzisi and colleagues [25] summarized the common components of these therapeutic programs that seem related to higher efficacy: starting as early as possible, which includes both early diagnosis and minimizing delays from diagnosis to treatment; being intensive; involving family; providing regular assessments to update treatment goals; providing supervision; encouraging spontaneous communication; supporting positive behaviors rather than tackling challenging behaviors; promoting skills via play with peers; and completing the acquisition, subsequent generalization and maintenance of new skills in natural contexts. These last two components are easier to achieve by including children into schools [26]. Despite these recent advances, many challenges remain, and the condition of individuals with ASD is still a severe burden [27].

Intellectual disability (ID) is frequently comorbid with ASD, although the exact rate depends on the specific subpopulation (e.g. [28, 29]). For example, when patients with both ASD and epilepsy are recruited, up to 25–45% of cases have ID [30]. ID is also a poor prognostic factor for long-term outcomes of ASD [31]. Very few intervention models to date have specifically addressed the comorbidity of ID with ASD, in particular when ID is moderate to severe. In addition, most clinical studies exploring treatment efficacy have excluded children with very low Intellectual Quotient (IQ) and/or associated disorders [3]. Peters-Scheffer et al. [32, 33] showed larger improvement on developmental age and adaptive skills in children with ASD and ID who received 2-years of low intensity behavioral treatment (LIBT) supplementing preschool services, compared to treatment as usual. Two randomized controlled trials compared 3-months of Pivotal Response Treatment (PRT) versus structured ABA in a school intervention setting for children with ASD and mild to moderate ID. They showed significant gains in social communication skills [34] and significantly lower levels of disruptive behavior [35] for PRT condition. PRT was also assessed for children with ASD and language delay: first, Minjarez and al [36] showed that parents can learn PRT in group therapy, resulting in correlated gains in children’s language; second improvements in language and cognitive functioning are maintained 3 months after completion of a 12-week PRT parent education group [37]. Finally, another study on minimally verbal children with autism showed that a brief, targeted intervention on joint attention and play (JASPER: Joint Attention Symbolic Play Engagement and Regulation intervention) can improve core deficits [38]. However, there is globally a dearth in the literature about specific interventions targeting children with ASD and ID. Furthermore, providing access to education is very difficult for this population. Thus, focusing research efforts on this understudied population who traditionally have been excluded from intervention trials is warranted whether it regards care as well as education.

In France, children with ASD and ID can be treated within special education centers (called “medico-social institutes”) and daycare hospitals where school activities represent a minimal part of daily activities. Rehabilitation take an important place and children spend a consequent amount of time receiving individual or group care dispensed by psychologists, speech or occupational therapists. This care often called “integrative care” is embedded within free play sessions with adults and peers, and educative socialized moments. This care organization lays on the idea that children with very low developmental levels have to build the basic social and communicational abilities, before being able to access formal instruction in a classroom. They are rarely included in mainstream classrooms and receive few formalized education.

However, these programs have not been well-studied, and the research that exists is mainly observational [39, 40]. The largest study included 152 French children with autism. They received a large panel of interventions, but these interventions were not described because the study focused on the developmental trajectories and the predictive factors of outcome [31]. French authorities, following the pressure of family associations, asked professionals to increase evidence-based research of French therapeutic programs for children with ASD and to promote education, which is less intensive compared to that of other European countries [41].

Indeed, mainstreaming is historically well-developed in Italy and is now common in many other countries. However, while education is compulsory in France, adapted education for children with ASD is not well-developed in France or in some other countries. This is particularly the case for children with severe ID, for whom mainstreaming might be anyway very difficult. In France, they are generally placed in daycare hospitals and medico-social institutes with very few educational support. Thus, few children with a low level of cognitive functioning can access adapted education. Could an individualized educational program, given by trained professionals in a one-to-one format, be implemented in these institutions, even for children with very low IQ? Could it bridge the gap between institutional care and adapted education in mainstream schools? How might this foster cognitive, socio-communicative and relational progress?

Thus a developmental and sequenced one-to-one educational intervention (DS1-EI) for school-aged children with ASD and low cognitive functioning was developed [42]. The intervention drew from several Narzisi principles (intensity, regular assessments, spontaneous communication, play with peers, supporting positive behaviors, providing supervision) and was based on 3 specific components. The first two are (1) capitalizing on teachers’ unique skills and (2) providing developmental and sequenced learning. Developmental learning implies that the focus of training is what is close to the expectations of a child’s development in a specific domain. Sequenced learning means that the teacher changes the learning activity every 10–15 min to maintain the child’s attention in the context of an anticipated time agenda. The third component is (3) supporting the child’s cognitive and communicative initiatives in a one-to-one condition with a different professional at each new activity [42]. The DS1-EI was designed to be implemented in French daycare hospitals or medico-educational institutes that usually include both individual care and milieu therapy.

A 3-year randomized, single-blind multicenter trial was implemented to assess DS1-EI efficacy [42]. Incorporating 10 h of DS1-EI could potentially not improve outcomes given the ongoing robust multimodal treatments given within the institutions. However, we expected a significant impact on core autism symptoms, developmental skills, and educational achievement based on the focus of the intervention. Here, we present the results of an interim analysis after 2 years of the DS1-EI intervention. The feasibility, acceptability, and mid-term efficacy of the DS1-EI intervention were evaluated.

## Methods

### Design and ethics

The study was a randomized, single-blind multicenter trial comparing the clinical course of 2 groups of children: the experimental group was exposed 4 mornings per week to a workshop class with an individualized, sequential and developmental pedagogy, completed with the usual institutional care during the remaining time (DS1-EI group); the control group was exposed full-time to the usual care of the institution (TAU group). Treatment as usual (TAU) was defined as all therapeutic interventions given to a specific child: this included various individual or group care according to patient’s needs given by psychologists, speech or occupational therapists, alternating with free play and educative sessions with adults and peers through various activities. In the TAU group, school time was not structured: teachers continued as usual to receive the children in their classroom for short individual or collective sessions. In general, they did not succeed in keeping the child attention for a long time. Thus, school sessions were most often reduced in time: E.g. at baseline, mean duration of school time was 3 h, meaning that TAU group received an average of 7 h of integrative care more per week compared to experimental group [42].

The trial duration was defined as 36 months but included 12-, 18- and 24-month intermediate assessments. The national health regulatory authority, *Agence nationale de sécurité du médicament et des produits de santé* (ANSM 130282B-31, April 16), approved the protocol; the local Ethics Committee, *Comité de Protection des Personnes* of the University Hospital Saint-Antoine, approved it as well on May 7, 2013. It was registered on the Australian New Zealand Clinical Trial Registry for public information availability (ACTRN12616000592448). The study adheres to CONSORT guidelines.

### Participant recruitment

All participants were recruited within French outpatient healthcare institutions specialized in treating children with ASD and ID. The children were already receiving a variety of treatments (the treatment as usual condition, TAU). Institutions were selected based on specific characteristics. The 11 sites were chosen to represent all French territories (including a Caribbean island). Each institution was required to dedicate a half-time special education teacher to the project for 3 years. Finally, institutions were recruited equally from the health sector (daycare hospital) and the medico-social sector (medico-educational institutes).

For informed consent, each parent was given by the local investigator an easily understandable information in the form of verbal explanations as well as an informational leaflet about the study. Randomization occurred only after written consent was obtained from the parents of potential participants.

The inclusion criteria were as follow: (1) age between 5 and 9 years old; (2) a current diagnosis of ASD confirmed by a clinical assessment based on ICD-10 criteria (International Classification of Diseases, 10th edition) and the Autism Diagnostic Interview-Revised (ADI-R) [43]; communication developmental age of 24 months and under or a 3-year speech delay based on a Vineland assessment; and determination by French education regulators that it was not possible to include the child in a mainstreamed or special education classroom.

Cognitive functioning was not directly addressed within the inclusion criteria, but children with autism and severe language delay or children who cannot be in mainstream classrooms generally have a low IQ or are not assessable. Children with known organic syndromes and/or unstabilized neuropediatric (e.g., seizures) or medical (e.g., diabetes mellitus) comorbidities were not excluded from the sample. During the medical assessment, we did, however, specifically list comorbidities. The exclusion criteria were limited only to parents’ refusal to participate and to families’ plans in the short term to change institutions for any reason.

Prior to randomization, children at each site had IQ assessments using the Kaufman Assessment Battery for Children, second edition (KABC II). As many children were not assessable through the KABC II, a developmental quotient (DQ) was calculated from the ratio of Vineland developmental age and chronological age. In accordance with the inclusion criteria that required a low level of communicative functioning and impossibility to be included in a mainstreamed or special education classroom, the observed level of functioning was generally very low (mean DQ was 30). Dyads of participants were matched by IQ or DQ and when possible by age and sex to minimize bias.

Randomization for group allocation was performed by drawing lots in each dyad per site, ensuring that each site would have 3 to 4 participants per DS1-EI group and TAU group. A methodological coordinating team at the Salpêtrière Hospital performed the randomization, thus making the process independent from the study sites. Table 1 lists inclusion sites, their location and their contribution to recruitment.

**Table 1:** Participants’ recruitment sites

Location	Type of institution	Number of patients
Amiens	IME	*N* = 6
Lille	DCH	*N* = 6
Aulnay sous Bois	DCH	*N* = 6
Argenteuil	IME	*N* = 6
Paris-Etincelle	DCH	*N* = 6
Paris-CEREP	DCH	*N* = 7
Sèvres	DCH	*N* = 8
Saint-Privas	IME	*N* = 8
Guadeloupe	DCH	*N* = 8
Marseille	IME	*N* = 6
Bagnols sur Cèze	IME	*N* = 8

*DCH* daycare hospital, *IME Institut medico-éducatif* (medico-educational institutes)

### Primary and secondary variables

**Fig. 1:**
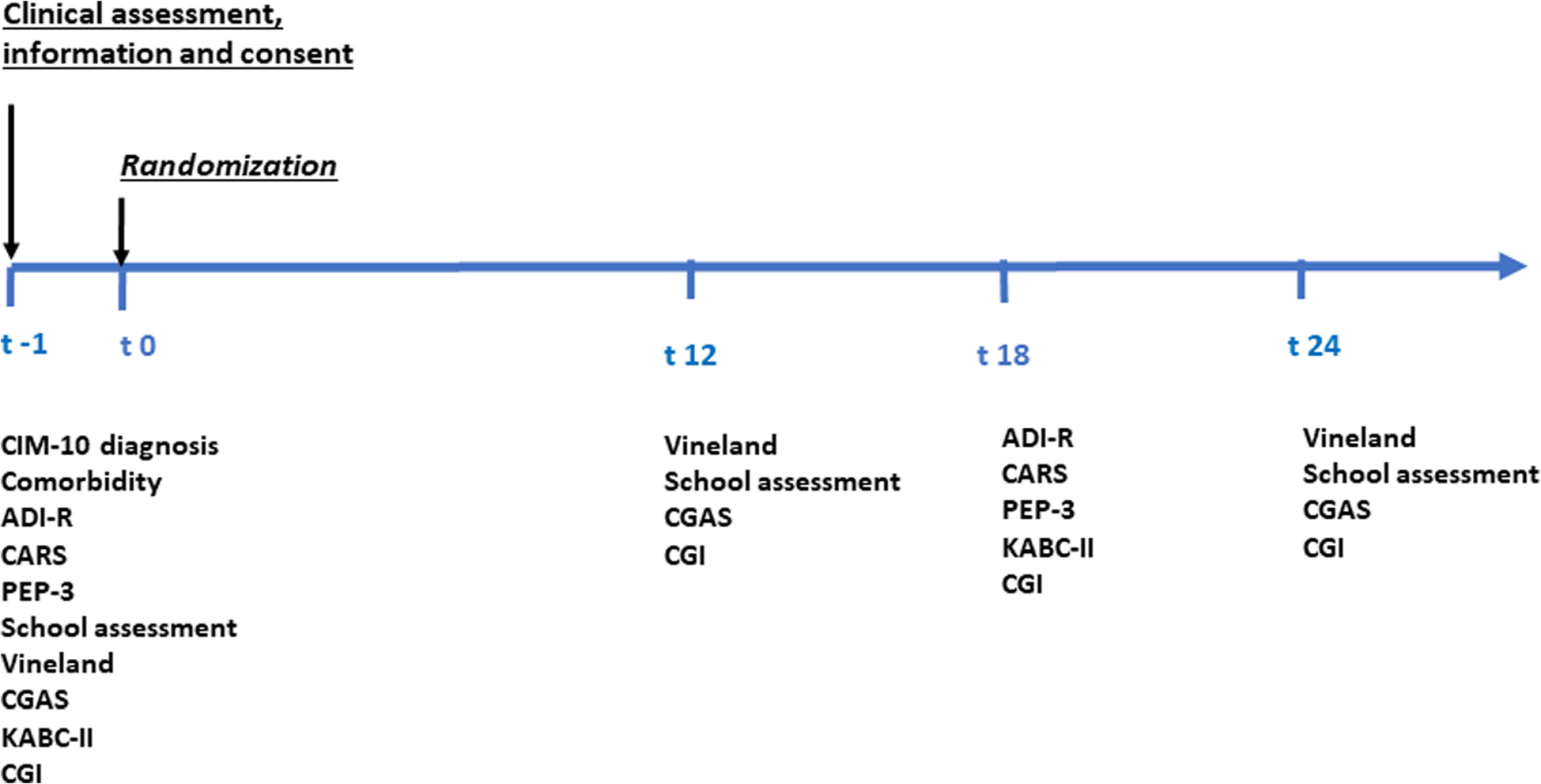
Schedule of assessments for the DS1-EI trial. ADI-R = Autism Diagnostic Interview-Revised; CARS=Childhood Autism Rating Scale; CGI=Clinical Global Impression; CGAS=Clinical Global Assessment Score; PEP-3 = Psychoeducational Profile, 3rd Edition; DQ = Developmental Quotient; KAB-C=Kaufmann Assessment Battery for Children

Figure 1 summarizes the variables and timing of measurement during the course of the trial. The primary outcome variables were (1) the Childhood Autism Rating scale (CARS) to measure autism severity [44]; (2) the Psychoeducational Profile, third edition (PEP-3), to measure the total DQ with 6 dimensional DQs related to expressive language, receptive language, cognition, fine motor skills, gross motor skills, and imitation [45] and (3) the school assessment based on French national abilities testing for preschoolers (http://eduscol.education.fr). Secondary variables included (1) the *Vineland Adaptive Behavior Scale II* (VABS-II) which is assessed via parent (as was the case in our study) or educator interview and used to determine children’s ability to perform daily activities required for personal and social autonomy. The VABS-II investigates four domains: Communication, Daily Living Skills, Socialization, and Motor Skills. Subscale scores are then totaled to yield a DQ [46]. (2) The *ADI-R*, which assesses interaction, communication and behavioral anomalies; (3) the Kaufman Assessment Battery for Children, second edition (*KABC-II)* standardized neuropsychological assessment of intelligence through measures of Verbal, Performance, Working Memory, Processing Speed and Total Quotients [47]; (4) the *Clinical Global Impression* (CGI), which assesses global symptom severity [48]; and (5) the *Clinical Global Assessment Scale* (CGAS) [49].

We evaluate clinical change during the 24-month period of the study using a single-blind procedure. Independent raters blind to study group allocation assessed participants for the main clinical assessments (PEP-3, KABC-II, CARS). For measures that required 2 weeks of participant observation (CGI, CGAS, school tests) or a parental interview (ADI-R, VABS-II), blind assessment was not feasible.

### DS1-EI principles

DS1-EI principles have been detailed in Tanet et al. (2016) [42]. It borrows some ingredients from ESDM (developmental expectation, positive engagement); from TEACCH (like adapted classroom environment or structured agenda); and from ABA (one-to-one condition, intensity, supporting positive behaviors rather than tackling challenging behaviors). Other aspects may resemble JASPER intervention (like following the child’s lead and interest in activities, responding to the child’s joint attention and requesting bids, matching child’s pacing and affect). Some aspects were also driven by a psychodynamic approach (like promoting children’s subjective and personal expression, whenever not expected, giving meaning to the child’s actions, helping the child to develop awareness of its own feelings). In Table 2, we summarize the major components. The intervention was based on individualized education taking place within small classrooms (3–4 children) with a teacher aided by the required number of assistants to reach one-to-one ratio. DS1-EI promotes social skills by alternating periods of social play with peers and sequences of one-to-one education tailored to each child’s developmental abilities. Teachers supported positive interactions and encouraged spontaneous communication with the children. Participants in the experimental group received the DS1-EI intervention for 4 mornings per week (2 h and 30 min per session); for the remainder of the week, they received the usual protocol of each site (other therapeutic practices as determined by each institution, e.g., speech therapy, occupational, therapy, social-skills group activities).

**Table 2:** A developmental and sequenced one-to-one educational intervention (DS1-EI) for autism spectrum disorder: main principles [36]

***Characteristics***	***Brief definition***
Setting	To be implemented in a small classroom with 3–4 pupils
	In an adapted environment
Intensive	One-to-one support 10 h per week in addition to other treatment practices (e.g., occupational therapy, speech therapy, psychotherapy)
Developmental	The focus of training is what is close to the child’s development within a domain
Sequenced	The 2.5-h sessions follow an anticipated and structured agenda
	Teachers change learning activities every 10–15 min to keep a child’s attention
Curriculum-based	A detailed assessment/curriculum is required to follow the developmental approach and to choose the appropriate cognitive/motor activity to be taught in each domain for preschoolers
Educational objectives	Given the developmental quotient of the children, the educational objectives are those of a second-grade program for preschoolers and include 4 domains (mathematics, language and communication, intermodality and autonomy)
Reinforcers	Supporting positive behaviors rather than tackling challenging behaviors
	Using positive emotion engagement from professionals
Group	Group activities are organized within the time schedule to encourage spontaneous communication and promote social skills through play with peers
Supervision	Regular supervision of teachers with updating of children’s educational objectives
Exploiting teachers’ unique skills	Implementation of the program will capitalize on teachers’ individual strengths, such as their knowledge of a specific method (e.g., the use of Picture Exchange Program) or of a particular child

**Fig. 2:**
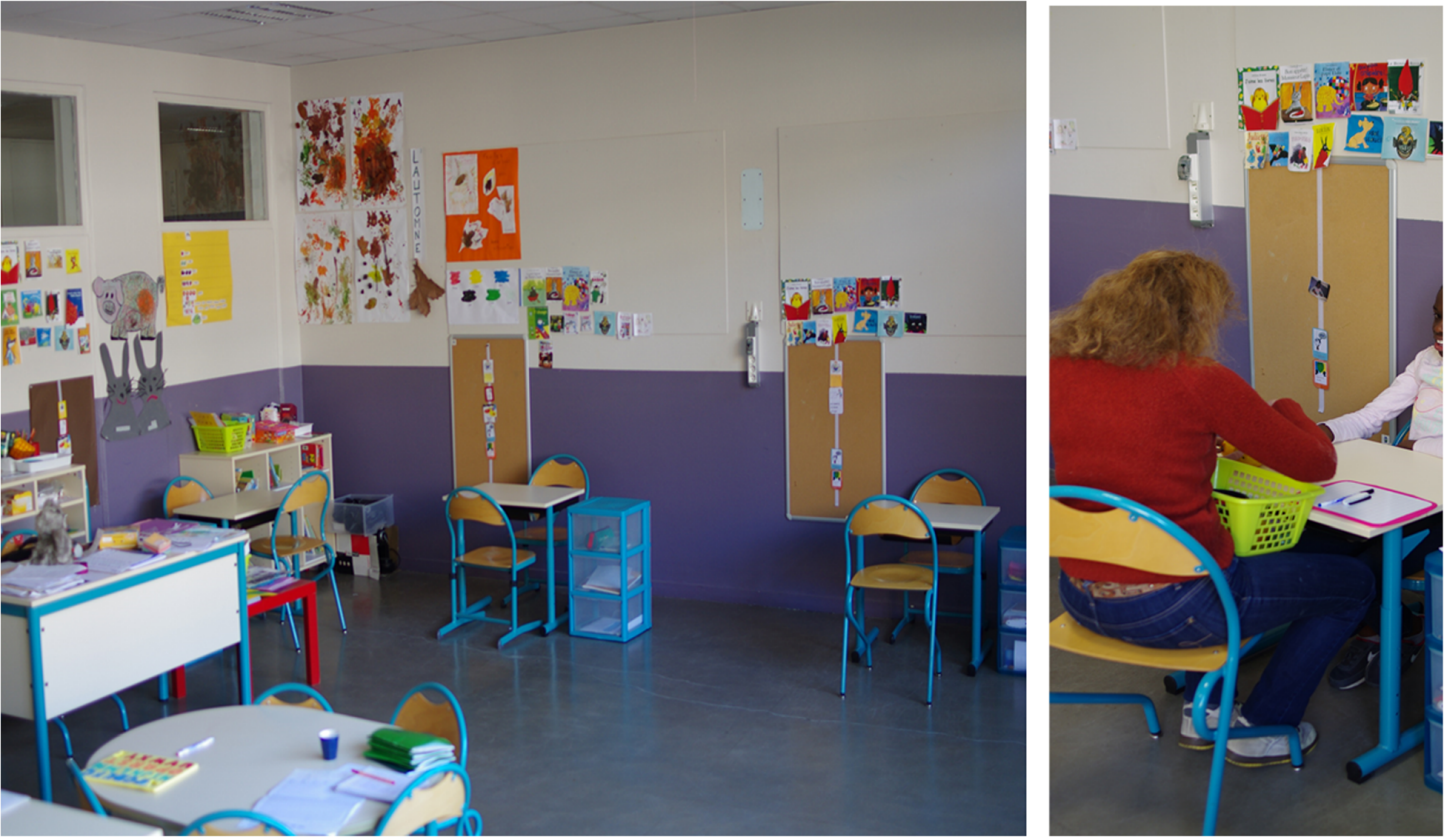
DS1-EI setting. **a** An example of one DS1-EI classroom: 1. Child’s and adult’s desk and chairs; 2. Child’s screen with pictograms; 3. The large table for mid-session group collaboration; (**b**) Each child is assigned a desk and two chairs (one for the child, one for the adult working with the child). During the learning sessions, the child sits with his back close to the wall. The adult working with the child sits facing the child. *Written informed consent was obtained from the parents for the publication of this image*

In France, teachers working in daycare hospitals and medico-educational institutes are specialized teachers belonging to the French public school system who are available to meet the needs of disabled children hosted in care institutions. Teachers usually receive in their classroom the children of the institution for short time sessions, individually or in small groups. For this study, they were trained to structure the classroom space and time session. The setting was a small classroom with 3 or 4 pupils, including a desk for each child, with two chairs (one for the child, one for the adult facing him to work with the child), a screen presenting pictures of the child’s schedule, and a locker. The child sat with his/her back close to the wall where the screen was placed (Fig. 2). The setting also included a large table for mid-session group snacks and an area with benches and carpets for the gathering sessions of all participants (both children and adults) with repetitive social activities at the beginning and end of a session.

The customized educational program followed developmental rules by focusing on the nearest expected activity or skill, given a child’s development in a particular area. Given that the program was curriculum-based, there were specific educational objectives for each child. The teacher created the curriculum and its objectives following academic recommendations from the French Ministry of National Education (http://eduscol.education.fr). Academic objectives involved four domains: language and communication, mathematics, intermodality, and autonomous behaviors. Because the DS1-EI by design follows a developmental approach, we performed a detailed assessment and curriculum for each child’s academic program. The curriculum allowed selecting appropriate cognitive/motor activities for training a given child within each above listed domain.

The morning session was structured in two ways. First, the 2.5 h session followed an expected, structured agenda that each child received on a screen. Second, every 10–15 min, professionals (teacher or assistants) were asked to change desks and activities. Thus, each 10–15 min, children had a new activity and a new teacher or assistant in order to maintain their attention, to challenge their need for sameness and to help generalize social and scholastic abilities. In sum, the structured context is meant to allow the child to be able to work with a variety of teachers and to experiment with new interpersonal relationships but in a rather predictable way.

Each classroom was overseen by a teacher aided by assistants as per the 1-to-1 program design. The assistants were specialized educators or nurses who care for the children inside the institution during the day, through other forms of intervention (individual or group approaches using various support methods and aiming to develop relational or instrumental abilities). All professionals (the teacher and the assistants) received a one-week session including: (1) an overview of the method (using positive affect, shared engagement, responsiveness and sensitivity to children’s cues); (2) specific instructions (focusing on verbal and nonverbal communication and supporting positive behaviors rather than tackling challenging behaviors). The DS1-EI detailed assessment/curriculum was explained, including how to best align learning proposals with a child’s particular developmental needs.

Supervision involved 2 different steps: 1) daily peer supervision sessions of verbal exchanges and written observations after class about each child in each domain with all professionals; 2) weekly supervision by a psychologist who gave support related to clinical issues, team clinical cohesion and proper adherence to DS1-EI implementation. To ensure fidelity of DS1-EI implementation and application over time, the main investigator (A H-B) came on each site at the beginning of the 3-year program and then at least 3 times per year to observe an entire morning session, to update each child’s academic objectives and to discuss any deviations for protocol or adjustments needed. Daily written observations were given to the main investigator. A formal fidelity grid was rated during the visits (see Additional file 2). In addition, an external audit examined on-site application of the protocol. They concluded that “*the observed homogeneity in program’s application shows that the on-site teams were engaged in an active process of formation and supervision to obtain a consistency in concrete intervention procedures as well as time and space structuring and individual strategies*”.

### Data control and statistical analysis

The number of patients to enroll was based on the following theoretical statistics estimation: for a moderate effect size (α = 0.6), a power fixed at 80%, and a level of significance for a *p*-value fixed at < 0.05, 80 patients randomized into two groups are required for a student *t*-test. Given our choice to use linear mixed models (see below) to take into account participant’s effect, we planned to recruit from 70 to 80 participants. The data were analyzed with the statistical program R, version 3.3.1 (R Foundation for Statistical Computing), using two-tailed tests and a level of significance set at 5%.

#### Deviations from protocol and missing data

The different populations were defined according to the type of deviation from protocol encountered. The “intent-to-treat” (ITT) population included all randomized participants, whatever the deviation status [minor deviation (e.g., could not attend DS1-EI full program for 1 week because of a flu) or major deviation (e.g. any cause of discontinuation and drop-out)]. The per-protocol (PP) population included only participants with no deviation or minor deviation. Missing data were monitored for each variable (number and percentage) and taken into account by the use of linear mixed-effects models. Note that school assessment was missing for a lot of TAU children at 24 months, precluding any group comparison for this variable.

#### Variables and covariates at baseline

Some initial characteristics of the population may influence the outcome. We compared all variables and covariates of the experimental (DS1-EI) group and the control (TAU) group at baseline to estimate the balance of the groups.

#### Variables and covariates at baseline

We compared all variables and covariates of the experimental (DS1-EI) group and the control (TAU) group at baseline. Quantitative variables were described using means and standard deviations and the difference between the two groups was tested by a Student test (Welch t-test) or a Wilcoxon rank-sum test according to distributional assumptions. Qualitative variables were described using n of occurrences and percentage and the intergroup differences were tested by a Chi-square test without continuity correction. When an expected count under the null hypothesis was less than 5, we used the Fisher exact test.

#### GLMM model

We used General Linear Mixed-effect Models to account for repeated measures (lme4 and lmer Test packages). Primary and secondary endpoints were used separately as a variable to be explained in the models. The effects were adjusted on the IQ/DQ score at baseline. All effects were fixed except the “subject” effect, which was a random effect. Explanatory variables in the model were DQ score at baseline, group, time, group*time interaction and subjects as random effect. The goal of assessing group*time interaction was to show whether change over time would differ between the two groups. To correct any bias, we planned to include covariates that showed an initial time imbalance in the multivariate model. Ultimately, none were required. For each judgment criterion, the *p*-values of group, time and group*time interaction effect were calculated, along with the endpoints delta and the estimated effect size using Cohen’s d formula for each group. The institutions entered the research in a phased manner (from June 2013 to June 2014), but within each of the 11 institutions, recruitment was done at all at once. Intermediate analyses were planned at 12, 18 and 24 months when all 11 institutions had completed each of these measures. Here, we present the results of these intermediate analyses.

## Results

### Feasibility and acceptability

**Fig. 3:**
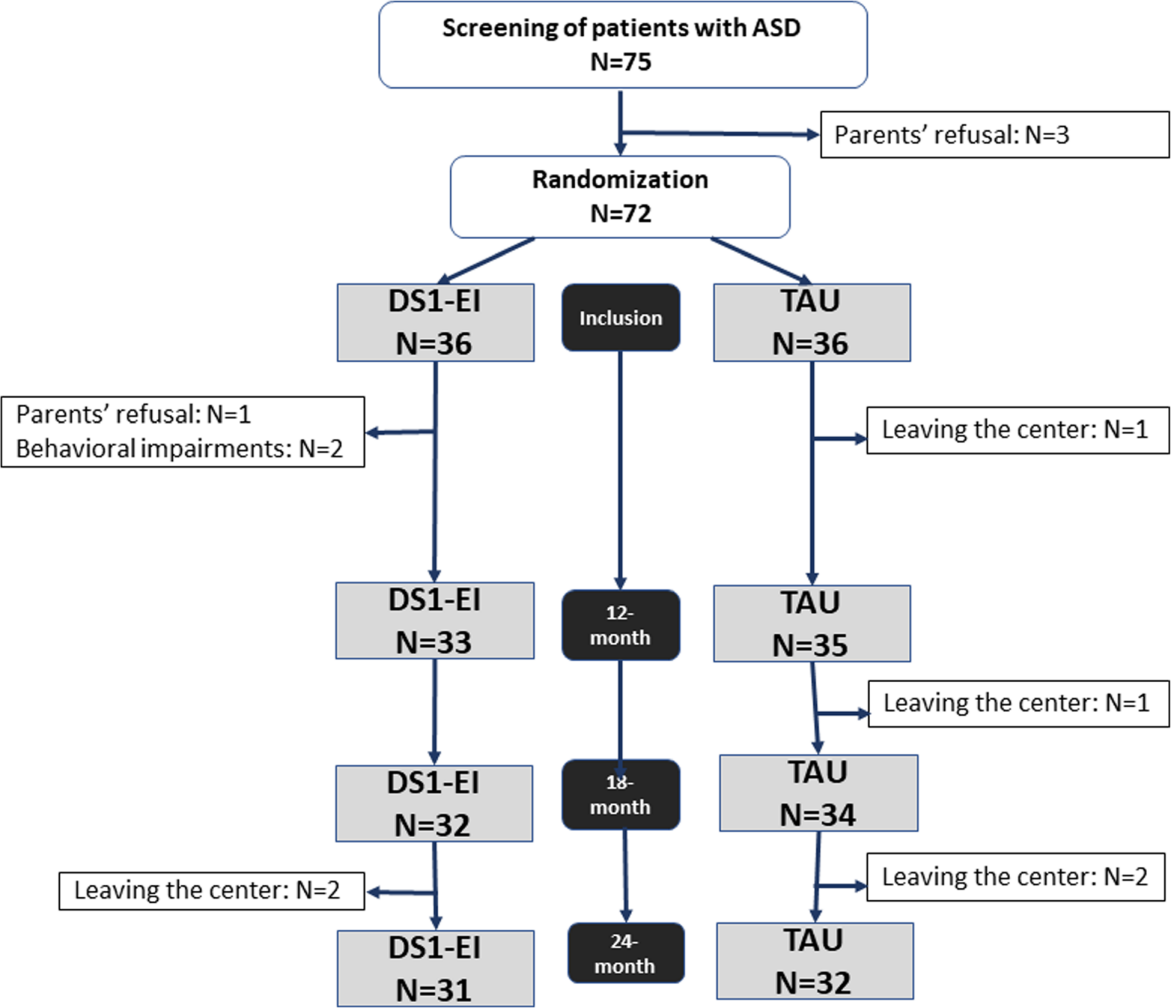
Diagram flow of the study

The diagram flow is shown in Fig. 3. A total of 75 participants were screened, but only 72 were included after randomization, as 3 were excluded. One family refused the randomization assigned to their child, which led further to the exclusion of the two patients of that initial pairing. One participant was recruited for inclusion and randomization, but as the institution was not able to find another child to form a pair for randomization, this participant was excluded. Of those 72 subjects in the ITT, 36 were randomized to each group. After randomization, 5 participants in the DS1-EI group and 4 participants in the TAU group left during the first 24 months of the study. The reasons for leaving included parents’ electing to withdraw their child from the study (*n* = 1), leaving the institution (*n* = 6) and extreme behavioral impairments preventing participation in school activities (*n* = 2). As evidenced by a good retention rate after 24 months (86.1 and 88.9% in the DS1-EI group and TAU group, respectively), the study was well accepted. We had an excellent rate of participation in the study, with only 4.2% of drop-outs attributable to children or parents electing to leave the study. In terms of institutional participation, feasibility was excellent as all sites included 6 to 8 participants. However, despite good implementation of the DS1-EI setting across institutions, some teachers did not understand that assessment of the intervention included administering the annual education achievement test to children from the TAU group. This misinterpretation will be corrected for the 36 months collection, but 24-months educational variables were unfortunately not obtained in all TAU participants. Thus, school variables were not interpretable at 24 months outcome due to missing data.

### Participants

Table 3 summarizes sociodemographic and clinical characteristics at baseline for the 72 randomized participants. No baseline differences between groups were found. The average age in both groups was approximately 7 years. As indicated from free text comments obtained in data collection, there was a large proportion of immigrant families, and many spoke little or no French. Further national and ethnic background information is not available due to strict restrictions in French law regarding this obtaining this type of data. Overall, participants had severe autism, with an average CARS greater than 40, and severe intellectual disability, as the mean Vineland developmental age in communication or socialization was approximately 15 months for a mean chronological age of 7 years. Due to study inclusion criterion of “determination by French education regulators that it is not possible to include the child in a mainstreamed or special education classroom,” baseline educational level could not be assessed, but may be described as “below primary school level.” Notably, this clinical population has received much less research attention since most treatment studies to date involved patients with much higher cognitive and functioning levels. In addition, 15 participants had 1–2 severe medical conditions (extremely preterm: *n* = 4; neonatal hypoxia: *n* = 1; genetic condition: *n* = 5 (Deletion of HNF1-B and TCF2 genes, Rubinstein-Taybi syndrome, William-Buren syndrome, Fragile X, Down syndrome); metabolic condition: *n* = 1; seizure: *n* = 1; hemiplegia: *n* = 1; cerebral malformation: *n* = 1; early puberty: *n* = 1; pigmentary retinitis: *n* = 1). The only difference we found at baseline between the 2 groups was in terms of the schooling variables, reflecting the study protocol: in the TAU group, children had very little schooling and were receiving an average of 3 h per week, compared to 10 h in the DS1-EI group.

**Table 3:** Sociodemographics and clinical characteristics at baseline

	DS1-EI group (***N*** = 36)	TAU group (***N*** = 36)	Test, p
*Sociodemographics*			
Sex: Female/Male	5 (13.9%) / 31 (86.1%)	6 (16.7%) / 30 (83.3%)	Chi2, *p* = 1
Age (in months)	82.4 (19.1)	87 (19.5)	W = 546.5, *p* = .26
Foreign language spoken at home (yes/no)	14 (38.9%) / 22 (61.1%)	18 (50%) / 18 (50%)	Chi2, *p* = .48
Associated disorder (yes/no)	6 (16.7%) / 30 (83.3%)	9 (25%) / 27 (75%)	Chi2, *p* = .56
Psychotropic medication (yes/no)	5 (13.9%) / 31 (86.1%)	6 (16.7%) / 30 (83.3%)	Chi2, p = 1
Education_(hours)	10 (3.3)	3.1 (4.3)	W = 1067.5, *p* < .001
Speech therapy^a^	0.8 (0.8)	0.8 (0.7)	W = 612, *p* = .83
Psychotherapy^a^	0.4 (0.6)	0.5 (0.7)	W = 619, *p* = .7
Psychomotricity^a^	0.7 (0.8)	1 (0.8)	W = 520, *p* = .12
Composite score of family support	2.2 (0.8)	2.1 (0.8)	W = 678.5, *p* = .72
Immigrant status (yes/no)	14 (38.9%) / 22 (61.1%)	18 (50%) / 18 (50%)	Chi2, p = .48
Composite parental education level	4.6 (1)	4.6 (1.2)	W = 362.5, *p* = .99
*Clinical characteristics*			
DQ	30 (10)	30 (10)	W = 692, *p* = .48
CARS	40.6 (7.1)	40.2 (7.1)	W = 681.5, *p* = .71
ADI-R interaction	20.8 (5.8)	20.1 (5.8)	W = 667, *p* = .67
ADI-R communication	11.8 (4.1)	10.8 (3.2)	W = 735.5, *p* = .22
ADI-R stereotypies	6.4 (2.7)	5.8 (3.2)	W = 693, *p* = .47
PEP-3 composite com	17.6 (7.1)	18.4 (7.9)	W = 600.5, *p* = .6
PEP-3 composite mot	24.6 (8)	25.7 (7)	W = 605, *p* = .63
PEP-3 maladaptive	9.7 (4.6)	9.6 (4.5)	W = 672.5, *p* = .79
VABS communication	15 (7.7)	15 (5.8)	W = 608.5, *p* = .66
VABS autonomy	28.6 (10.6)	27.9 (10.5)	W = 682, *p* = .71
VABS socialization	15.2 (8.2)	14.6 (9)	W = 704.5, *p* = .53
VABS motricity	33.3 (10.1)	32 (9.8)	W = 697.5, *p* = .58
CGAS	25.8 (12)	24.8 (11)	W = 696.5, *p* = .59

*DS1-EI* Developmental and Sequenced One-to-One Educational Intervention, *TAU* Treatment as usual, *DQ* Developmental Quotient according to Vineland Developmental age relative to chronological age, *ADI-R* Autism diagnostic interview-revised, *PEP-3* Psycho-educational profile, 3rd Edition, *VABS* Vineland adaptive behavior scale, *CGAS* Clinical global assessment score

^a^Mean number of session per week per participants

### Intent-to-treat (ITT) outcomes at 18 and 24 months (Figs. 4, 5)

The ITT analyses were conducted on all randomized participants (*n* = 72). The primary variables (namely, CARS and PEP composite scores) were measured at 18 months and were the only blind variables. Intermediate educational variables are not presented here because they have not been assessed/scored in all patients at this time. The 24-month ADI-R scores were not blind, as parents were aware of group allocation. Table 4 summarizes the changes over time and the effect sizes for each variable by group. We found no significant difference between the two groups on the primary variables and no significant difference on improvement in the group*time interaction. In contrast, there was a significant time effect for all scores (ADI-R Interaction, CARS, PEP-communication, PEP-Motricity, PEP-Maladaptive scores). Because there was no group*time interaction, we conclude that both groups significantly improved over time. In Table 4, we present *p*-values from the model, and for each group, score’s deltas and associated effect sizes, which were generally moderate to strong, ranging between 0.5 and 0.71.

**Fig. 4:**
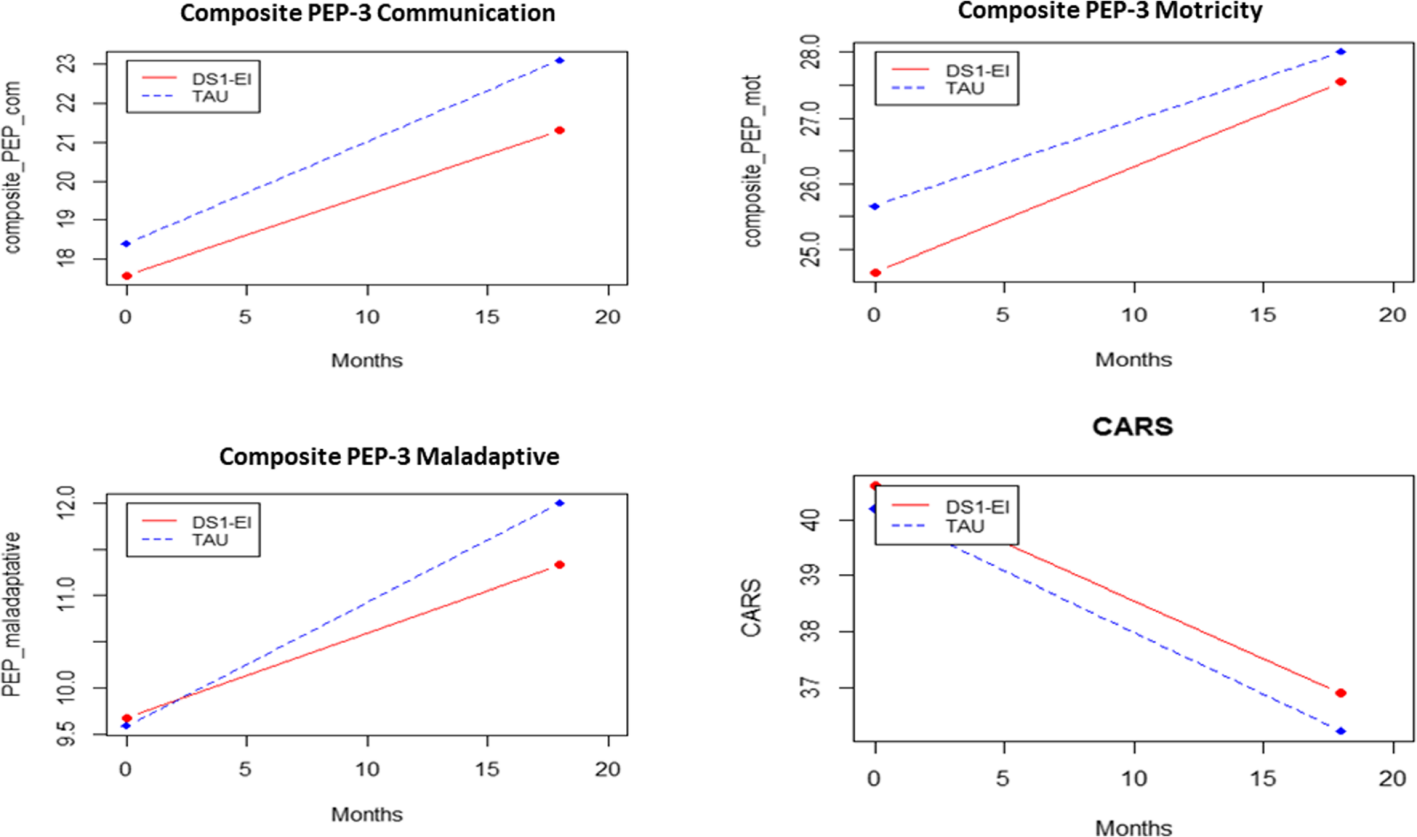
Changes in primary variables from baseline to 18 months in the DS1-EI and the TAU groups. DS1-EI: Developmental and Sequenced One-to-One Educational Intervention; TAU: Treatment as usual; CARS=Childhood Autism Rating Scale; PEP-3 = Psychoeducational Profile, 3rd Edition

**Fig. 5:**
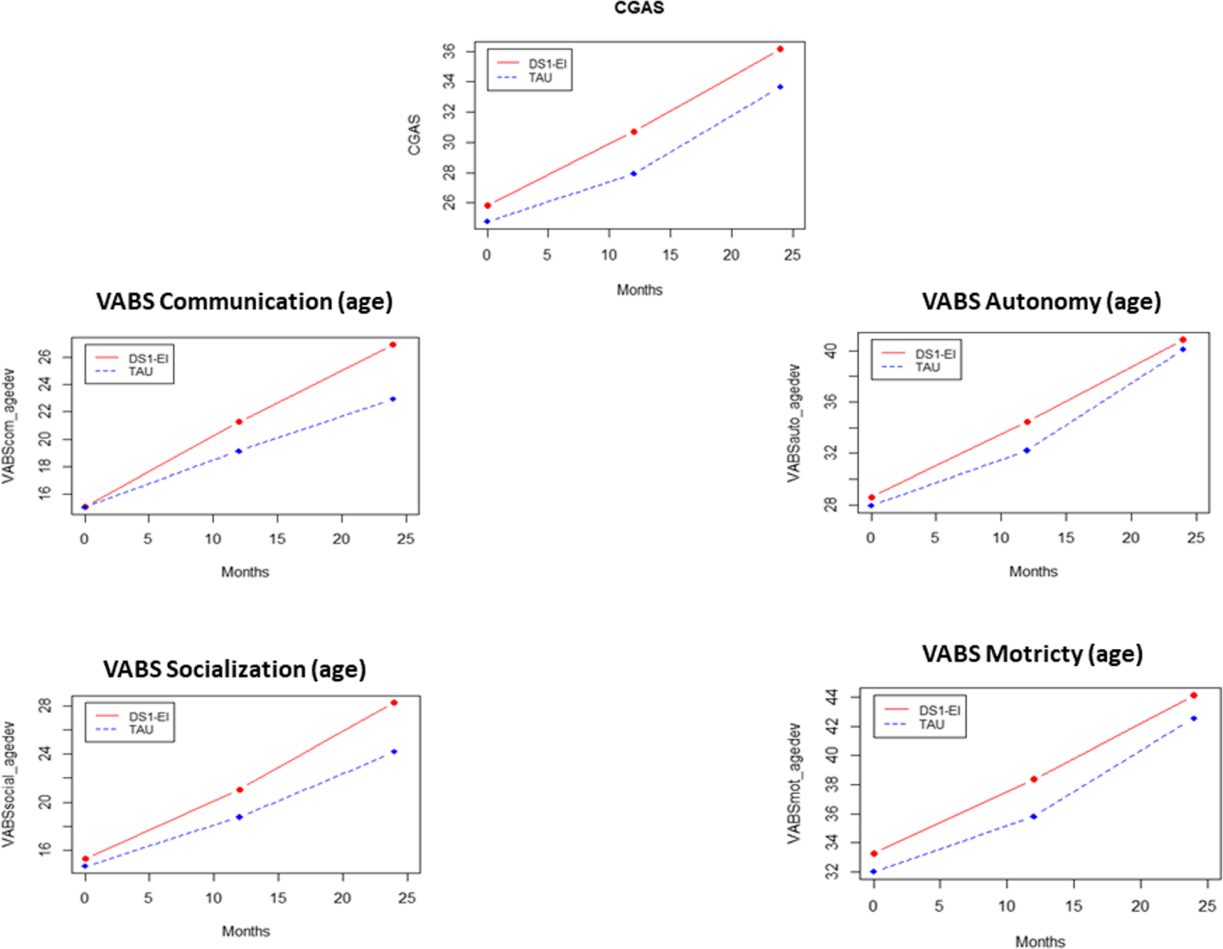
Changes in secondary variables from baseline to 12 and 24 months in the DS1-EI and the TAU groups. DS1-EI: Developmental and Sequenced One-to-One Educational Intervention; TAU: Treatment as usual; VABS: Vineland adaptive behavior scale; CGAS: Clinical global assessment score

**Table 4:** Intent-to-treat analysis outcomes at 18 months

	∆ DS1-EI	ES DS1-EI	∆ TAU	ES TAU	P (time)	P (group)	P (group*time)
CARS	−3.7	0.52	−4.0	0.64	.003	.580	.756
ADI-R interaction	−2.9	0.52	−3.0	0.48	.016	.449	.688
ADI-R communication	−0.9	0.19	−0.7	0.19	.421	.234	.781
ADI-R stereotypies	−0.5	0.25	0.4	0.21	.322	.325	.161
PEP communication	3.7	0.71	4.7	1.01	.000	.387	.271
PEP motricity	2.9	0.55	2.4	0.59	.001	.315	.805
PEP maladaptive	1.7	0.49	2.4	0.90	.006	.776	.150

*CARS* Child autism rating scale, *ADI-R* Autism diagnostic interview-revised, *PEP* Psycho-educational profile. *P* values comes from the GLMM. However, in order to show the changes in each group, this table includes the score variation between 0 and 18 months with its corresponding effect size

The variables measured at 12 and 24 months were not blind, as they were based on parental or professional interviews. All secondary variable results were consistent with the above reported results. We found no group*time interaction, indicating no significant difference between groups in score change over time. However, there was a significant effect time for all Vineland scores and for the CGAS, reflecting a significant improvement over time in both groups. Again, effect sizes of score change between 0 and 24 months were rather strong, ranging from 0.75 to 1.18. The overall results are presented in Table 5.

**Table 5:** Intent-to-treat analysis outcomes at 12 and 24 months

	∆ DS1-EI	ES DS1-EI	∆ TAU	ES TAU	P (time)	P (group)	P (group*time)
VABS communication	11.9	0.85	7.9	0.91	<.001	.610	.306
VABS autonomy	12.2	0.93	12.2	1.06	<.001	.736	.500
VABS social	13.0	0.75	9.6	0.91	<.001	.738	.661
VABS motor	10.9	0.89	10.6	1.00	<.001	.942	.448
CGAS	10.3	1.18	8.9	1.27	<.001	.928	.851

*VABS* Vineland adaptive behavior scale, *CGAS* Clinical global assessment score

*P* values comes from the GLMM including 12 months intermediate assessments. To show the changes in each group, this table includes the score variation between 0 and 24 months with its corresponding effect size

### Per-protocol outcomes at 18 and 24 months (supplementary material)

Per-protocol analyses were conducted on participants who remained in the study during the entire 24 months (the end point of intermediary analysis). As 9 patients dropped out during the first 24 months of the study (*n* = 5 in DS1-EI, *n* = 4 in TAU), the PP analysis included 63 patients (*n* = 31 in DS1-EI, *n* = 32 in TAU).

PP analysis for primary variables measured at 18 months showed similar results as those from the ITT analysis. There was no significant group effect at baseline and no significant effect in the group*time interaction. However, there was a significant time effect. Nearly all variables (the CARS, the ADI-R interaction and the 3 scores of the PEP: motricity, communication, adaptation) showed a significant improvement over time in both groups.

For the secondary variables measured at 18 and 24 months, the results yielded a similar effect in the PP analysis as in the ITT analysis. There was no significant group effect at baseline and no significant group*time interaction effect. However, almost all variables (Vineland scores and CGAS at 12 and 24 months) showed a significant improvement over time in both groups.

## Discussion

### Summary of the results

Here we report the interim results at 24 months of the 36-month randomized controlled trial testing the use of DS1-EI as a relatively intensive educational treatment provided in small classrooms through 2.5-h sessions and under regular supervision. We included 36 children with ASD and ID in the experimental DS1-EI group and 36 matched controls. There was a significant improvement in primary and secondary variable scores in both groups. In addition, there was excellent patient and family participation in the study, with a limited number of patients lost to follow-up (12.5%). Acceptability and sustainability are high for both the institutional teams and for the children. This indicates that participation in the experimental intensive schooling group is feasible for both teachers and children despite patients’ low IQ.

However, the primary and secondary outcomes of the trial were negative. At endpoint, we found no significant change for interaction between time and group. We found a significant improvement in both groups (i.e., exposed to DS1-EI or TAU) by time with moderate-to-large effect sizes for CARS, PEP-3, VABS and CGAS scores. This indicates that TAU and DS1-EI in daycare hospitals and special education facilities improve children in similar ranges. Thus, we don’t know if spending 10 h a week in a classroom with one-to-one support add much instead of receiving the usual care. Even if families in general request more time in classroom for their children [41], the extra cost is not supported at 24-months follow-up. The lack of a significant group effect may also mean that the DS1-EI children have improved their abilities to be in a classroom but are not able to generalize in another context yet. The context in which outcome measures are assessed influences the reported magnitude of effects. A review of 23 studies suggested that those using measurement contexts highly similar to the treatment context, had an 82% probability of a positive treatment effect, compared to a 33% probability for studies that used generalized measures [50]. Unfortunately, our contextual measure (school assessment) could not be reported at 24-months due to missing data. Every effort will be done to recall teachers of children in the TAU group to report school assessment at 36-months.

Other possible explanations for the recorded improvement in performance over 24 months in both groups may be the children’s maturation and test-retest advantage. Lastly, the lack of significant DS1-EI effects may be due to the too-early measurements (at 24 months instead of at the study conclusion at 36 months). For example, in the UK’s Preschool Autism Communication Trial studies, significant improvement was shown only at the 5-year follow-up [15, 51]. Indeed, changes in ASD symptoms are not easy to measure, and there is still no consensus on the best variable to be used in a randomized controlled trial [52]. As we know that a low IQ is a factor of poor prognosis, the progresses may be very slow and subtle. Using a direct, observational measure of social communication abilities (instead of the Vineland) may have yielded different information. For example, Fuller (2019), in a systematic review of the literature including 29 studies reported numerous significant effects on tools directly targeting social communication outcomes. Finally, another limit is the moderate size of the sample in regard to the heterogeneity of the children. Notably, children with comorbid conditions were not excluded from our study.

### Implications of results

Although the trial was negative, the results have some interesting implications, especially in the French context. First, the evaluation of institutional programs for children with ASD and ID during an evidence-based study is partially in response to families’ request [53, 54]. Given the long duration (36 months) of the entire study, and the required subsequent time needed to analyze trial effectiveness, we did not change children’s therapeutic protocol in the TAU group during the study period. Children in both groups continued to receive standard daycare hospital or *Institut Médico-Educatif* (special education center) treatments, and including individual and group educational activities, regular sessions of speech therapy, psychotherapy, and occupational therapy [42]. The range of improvement seen in this study was similar to that obtained in the 3-year follow-up observational study conducted by Baghdadli et al. [31] in France.

Second, the study results are salient in the current French context. Since the seventies, France has developed specialized institutions for children with ASD. Daycare hospitals and medico-social institutes provide various integrative care aiming to develop socio-communicative abilities through play activities in a developmental view of building social relationships. But they offer very few educational support. However, French authorities have enshrined in the legislation the right for disabled children to be included in mainstream schools [54]. Thus, special settings have been developed in schools and French children are more and more included in school. However, many children with severe ASD and ID initially cannot be placed in mainstream or even special education classrooms, especially if significant behavioral problems are present. Meanwhile, French families are still advocating to have more children with ASD included in mainstream schools even if there is comorbid moderate-severe ID [53, 54]. Several agencies, in France and in other countries, have recommended conducting interventions in school-based settings. Research demonstrates that children with ASD benefit from being with their peers in a school setting, and a variety of interventions could be used in classrooms [55–57], yet there is a gap between this research and educational practice [58]. The context of a preschool seems to provide opportunities to develop communication skills [59], and by offering children opportunities to enter into play groups, teachers can reinforce a nonverbal ASD child’s requests [60]. Intervention in schools may be more generalizable because students can use learned communication skills within a natural environment such as a classroom [61]. Two objectives should be combined: improving core deficits and practicing social communication [62]. To date, more research is needed to identify the most effective classroom interventions [63]: the majority of studies examine behavioral interventions in school [56, 64], and the literature on school-based interventions generally deals with the question of providing intervention in school [64, 65]. They do not explore which ingredients will allow children with severe disabilities to rise to a learner position and enter school effectively. DS1-EI is one approach to allow children with autism and severe ID to participate in school activities, while developing socio-communicative abilities simultaneously. Impossibility of inclusion in an external school was an inclusion criterion. Through this setting, children who had very low abilities and often severe behavioral disorders and who were thus initially receiving very few time of unstructured education were actually able to participate in structured school activities 10 h per week. DS1-EI may be integrated into daycare hospitals or special education institutes, used to prepare children to enter mainstream schools, or delivered within the school context.

However, to determine full results for both clinical and educational assessments, the study will need to continue until the 36-month endpoint. Even if the effects of DS1-EI do not generalize, a greater improvement in educational variables in DS1-EI is expected than in TAU since educational achievement is the main target of the experimental treatment. In the final year of the study, assessing educational variables for both groups will be a priority in order to have valid data available for comparison across both groups.

### Strengths and limitations

Strengths of this study are a design of multicentre randomized, single-blind controlled trial and the focus on an understudied sample of children with autism and comorbid moderate/severe intellectual disability and no exclusion of children with medical conditions. As limitations, the inclusion of children with comorbidities might have limited outlining differential effects according to the specific intervention, as sample size do not allow subgroup analyses (e.g. without comorbid conditions). However, we believe that these children are relevant to study because it is the population we are dealing with. Second, these 24-month interim analyses did not include scholar assessment, an important target of the specific intervention, and might be a too short duration to differentiate DS1-EI from TAU. We expect to answer this limitation with the end-point analysis at 3 years that we will perform within the coming year. Third, randomization was conducted on paired dyads in each site to limit recruitment biases. However, given the duration of the study, we cannot exclude that contamination took place in TAU by implementing DS1-EI in each site as teachers talk to each other between lessons or after lessons, and discuss things that are successful in their classes, or about problems they encounter. By doing so they may have adopted ingredients from DS1-EI to TAU. Last, recruitment was confined to France.

## Conclusions

We implemented a new intervention, DS1-EI, in children aged 5 to 9 with cooccurring ASD and ID. This program was designed to increase children educational achievement in French daycare hospitals or medico-educational institutes. The study did not show that DS1-EI was superior to TAU in treating children with ASD and ID within 24 months. However, the low dropout rate shows that within structured and integrative settings, providing more educational instruction than is the usual practice in France is feasible. As the current report is an interim analysis, we plan to assess at our study end-point (36 months) whether a total of 3 years of the DS1-EI intervention will show a significant difference compared with TAU. We will also determine which initial factors may predict better outcomes.

## Supplementary information

**Additional file 1. **Supplementary material DS1-EI 24 Months BMC Pediatrics. Per protocol analysis of the DS1-EI randomized controlled trial at 24 months. **Table S1.** Comparison at baseline (per protocol population, *N* = 63). Clinical characteristics at baseline of the per-protocol population by group. **Table S2.** Variables at 18-month outcome (PP population, *N* = 63). Changes according to time for CARS, ADI-R and PEP-3, by group. **Table S3.** Variables at 12- and 24-month outcome (PP population, *N* = 63). Changes according to time for VABS and CGAS, by group. **Figure S1.** Variables at 18-month outcome (PP population, *N* = 63). Changes according to time for CARS and PEP-3, by group. **Figure S2.** Variables at 12- and 24-month outcome (PP population, *N* = 63). Changes according to time for VABS and CGAS, by group.

**Additional file 2.** DS1-EI fidelity grid rated during the study supervision.

## Data Availability

Data can indirectly be traced back to the study participants. According to French and EU personal data legislation, access can only be made upon request. The request should in this case be addressed to the corresponding author David Cohen, and will be handled on a case by case basis.

## Abbreviations

ASD: Autism spectrum disorder
ID: Intellectual disability
DS1-EI: Developmental and Sequenced One-to-One Educational Intervention
TAU: Treatment-as-usual
CARS: Childhood Autism Rating scale
PEP-3: Psychoeducational Profile, third edition
VABS-II: Vineland Adaptive Behavior Scale II
CGAS: Clinical Global Assessment Scale
ITT: Intent-to-treat
PP: Per-protocol
ANSM: *Agence nationale de sécurité du médicament et des produits de santé*
TEACCH: Treatment and Education of Autistic and Communication Handicapped Children
ABA: Applied Behavioral Analysis
PRT: Pivotal Response Training
ESDM: Early Start Denver Model
DIR: Developmental, Individual-Differences and Relationship-based Model
PACT: Parent-mediated social communication therapy
EIBI: Early intensive behavioral intervention
IQ: Intellectual Quotient
ICD-10: International Classification of Diseases, 10th edition criteria
ADI-R: Autism Diagnostic Interview-Revised
DQ: Developmental Quotient
KABC-II: Kaufman Assessment Battery for Children, second edition
CGI: Clinical Global Impression
