# Supplementary material for: Developmental and sequenced one-to-one educational intervention (DS1-EI) for autism spectrum disorder and intellectual disability: a two-year interim report of a randomized single-blind multicenter controlled trial
Source: BMC Pediatr. 2020 May 29;20:263. doi: 10.1186/s12887-020-02156-z (PMC7260851; doi:10.1186/s12887-020-02156-z)
Supplement: Supplementary file 1 — Additional file 1. Supplementary material DS1-EI 24 Months BMC Pediatrics. Per protocol analysis of the DS1-EI randomized controlled trial at 24 months. Table S1. Comparison at baseline (per protocol population, N = 63). Clinical characteristics at baseline of the per-protocol population by group. Table S2. Variables at 18-month outcome (PP population, N = 63). Changes according to time for CARS, ADI-R and PEP-3, by group. Table S3. Variables at 12- and 24-month outcome (PP population, N = 63). Changes according to time for VABS and CGAS, by group. Figure S1. Variables at 18-month outcome (PP population, N = 63). Changes according to time for CARS and PEP-3, by group. Figure S2. Variables at 12- and 24-month outcome (PP population, N = 63). Changes according to time for VABS and CGAS, by group. [file 12887_2020_2156_MOESM1_ESM.docx]

# Supplementary material

# Per protocol analysis of the DS1-EI randomized controlled trial at 24 months

## 1. Table S1: Comparison at baseline (per protocol population, N=63)

|  | DS1-EI group (N=31) | TAU group (N=32) | Test, p |
| --- | --- | --- | --- |
| DQ | 30 (10) | 30 (10) | W=692, p=0.48 |
| CARS | 40.4(7) | 40.4(6.5) | W=565, p=0.8 |
| ADI-R interaction | 20.7(5.8) | 20(6.1) | W=440, p=0.76 |
| ADI-R communication | 11.4(3.5) | 11(3.1) | W=470, p=0.44 |
| ADI-R stereotypies | 6.2(2.6) | 5.5(3.2) | W=473.5, p=0.41 |
| PEP-3 composite com | 17.9(7.3) | 18.3(7.9) | W=538.5, p=0.78 |
| PEP-3 composite mot | 25.2(8) | 25.8(7) | W=544.5, p=0.84 |
| PEP-3 maladaptive | 9.9(4.5) | 9.4(4.2) | W=613, p=0.52 |
| VABS communication (age) | 15.2(8.4) | 14.2(5.9) | W=417, p=0.68 |
| VABS autonomy (age) | 30(11.2) | 27.2(10) | W=453, p=0.32 |
| VABS socialization (age) | 15.7(8.2) | 13.6(8.9) | W=467.5, p=0.21 |
| VABS motricity (age) | 34(10) | 30.9(9.3) | W=466.5, p=0.22 |
| CGAS | 28.7(10.8) | 25.2(10.8) | W=363, p=0.21 |

DS1-EI: Developmental and Sequenced One-to-One Educational Intervention; TAU: Treatment as usual; DQ: Developmental Quotient according to Vineland Developmental age relative to chronological age. ADI-R: Autism diagnostic interview-revised; PEP-3: Psycho-educational profile, 3^rd^ Edition; VABS: Vineland adaptive behavior scale; CGAS: Clinical global assessment score.

*Mean number of session per week per participants

## Table S2: Variables at 18-month outcome (PP population, N=63)

|  | ∆ DS1-EI | ES DS1-EI | ∆ TAU | ES TAU | p time | p group | p group*time |
| --- | --- | --- | --- | --- | --- | --- | --- |
| CARS | -3.8 | 0.52 | -4.2 | 0.64 | 0.004 | 0.630 | 0.808 |
| ADI-R Interaction | -3.3 | 0.60 | -2.9 | 0.48 | 0.008 | 0.214 | 0.984 |
| ADI-R Communication | -0.3 | 0.10 | -0.9 | 0.19 | 0.839 | 0.495 | 0.477 |
| ADI-R Stereotypies | -0.6 | 0.24 | 0.7 | 0.21 | 0.308 | 0.251 | 0.107 |
| Composite PEP-3 com | 3.9 | 0.71 | 4.8 | 1.01 | 0.000 | 0.392 | 0.344 |
| Composite PEP-3 mot | 2.6 | 0.49 | 2.3 | 0.59 | 0.005 | 0.322 | 0.981 |
| PEP-3 maladaptive | 1.5 | 0.44 | 2.6 | 0.90 | 0.020 | 0.970 | 0.079 |

CARS: Child autism rating scale; ADI-R: Autism diagnostic interview-revised; PEP-3: Psycho-educational profile, 3rd Edition. P values comes from the GLMM. However, in order to show the changes in each group, this table includes the score variation between 0 and 18 months with its corresponding effect size.

###

### Table S3: Variables at 12- and 24-month outcome (PP population, N=63)

|  | ∆ DS1-EI | ES DS1-EI | ∆ TAU | ES_TAU | p_time | p_group | p_group*time |
| --- | --- | --- | --- | --- | --- | --- | --- |
| VABS communication (age in months) | 12.2 | 1.08 | 8.7 | 0.91 | 0 | 0.539 | 0.196 |
| VABS autonomy (age in months) | 12.1 | 1.30 | 12.9 | 1.06 | 0 | 0.845 | 0.661 |
| VABS socialization (age in months) | 12.2 | 0.93 | 10.6 | 0.91 | 0 | 0.823 | 0.859 |
| VABS motricity (age in months) | 12.6 | 1.17 | 11.7 | 1.00 | 0 | 0.847 | 0.888 |
| CGAS | 8.8 | 1.19 | 8.4 | 1.27 | 0 | 0.541 | 0.904 |

## VABS: Vineland adaptive behavior scale; CGAS: Clinical global assessment score.

## P values comes from the GLMM including 12 months intermediate assessments. To show the changes in each group, this table includes the score variation between 0 and 24 months with its corresponding effect size.

## Figure S1: Variables at 18-month outcome (PP population, N=63)

DS1-EI: Developmental and Sequenced One-to-One Educational Intervention; TAU: Treatment as usual; PEP-3: Psychoeducational Profile, 3^rd^ Edition; CARS: Childhood Autism Rating Scale

| PEP-3 composite Communication  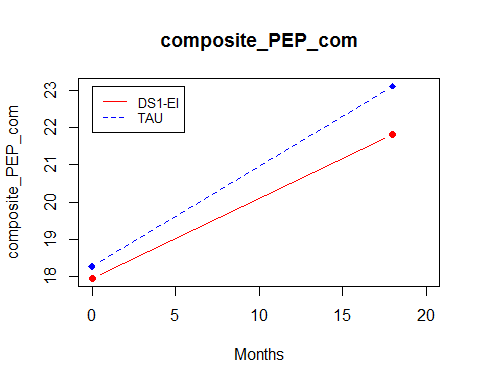 | PEP-3 composite Motricity  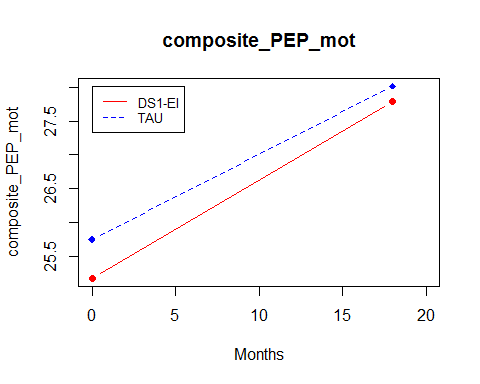 |
| --- | --- |
| PEP-3 Maladaptive  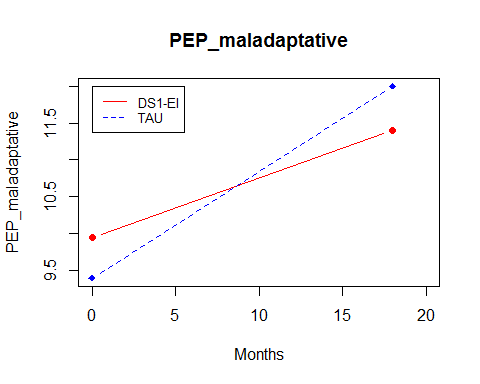 | 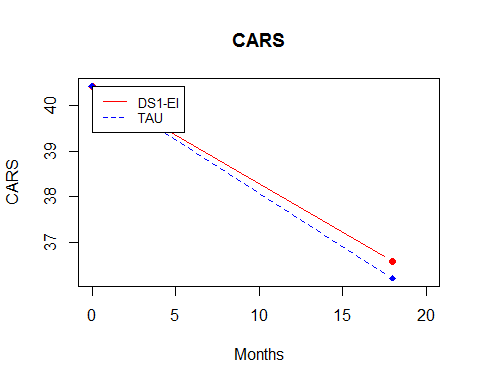 |

### Figure S2: Variables at 12- and 24-month outcome (PP population, N=63)

DS1-EI: Developmental and Sequenced One-to-One Educational Intervention; TAU: Treatment as usual; VABS: Vineland adaptive behavior scale; CGAS: Clinical global assessment score

| VABS Communication (age in months)  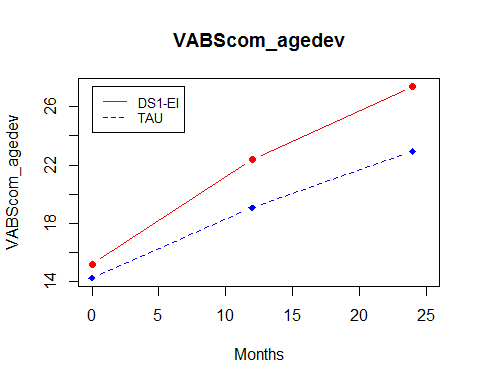 | VABS Autonomy (age in months)  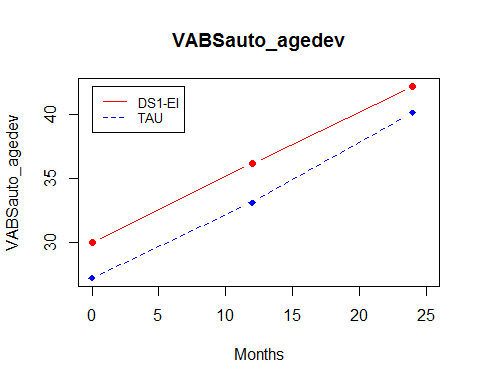 |
| --- | --- |
| VABS Socialisation (age in months)  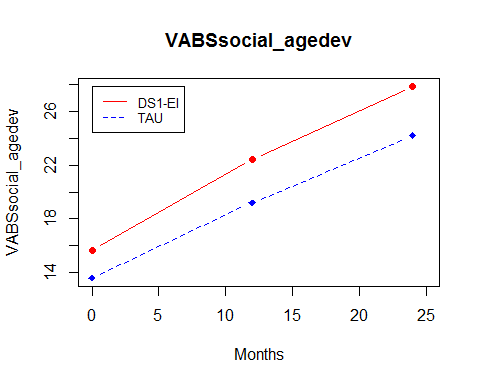 | VABS Motricity (age in months)  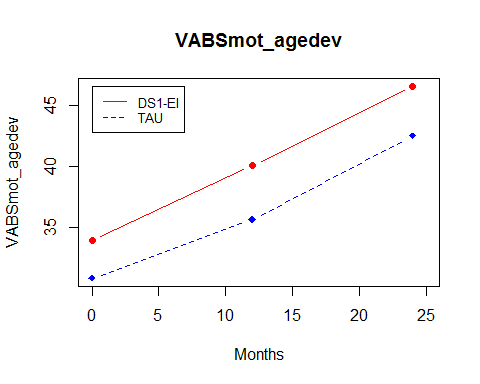 |
| 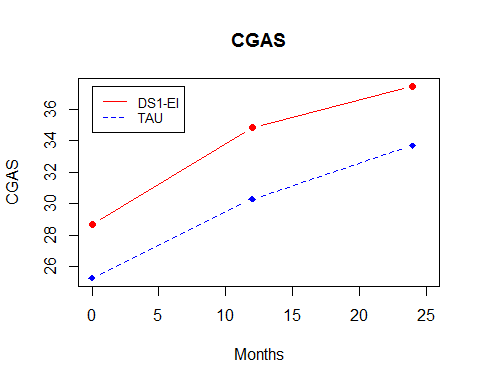 |  |
