# Supplementary material for: Developmental and sequenced one-to-one educational intervention (DS1-EI) for autism spectrum disorder and intellectual disability: a two-year interim report of a randomized single-blind multicenter controlled trial
Source: BMC Pediatr. 2020 May 29;20:263. doi: 10.1186/s12887-020-02156-z (PMC7260851; doi:10.1186/s12887-020-02156-z)
Supplement: Supplementary file 2 — Additional file 2. DS1-EI fidelity grid rated during the study supervision. [file 12887_2020_2156_MOESM2_ESM.docx]

**Annex 1 : DS1-EI fidelity grid rated during the study supervision**

Each criteria were assessed during the supervision in each site. Here, a cumulative rating is given for each site and criteria (see meaning below).

| Location and N* | C1 : Dayly regularity | C2 : Duration | C3 : DS1-EI stability staff | C4 : Number of activities | C5 : Notes and reports per week | C6 : Implication of the principal investigator | C7 : Adaptation of the activities according to developmental curriculum | C8 : Ability to take into account remarks after supervision |
| --- | --- | --- | --- | --- | --- | --- | --- | --- |
| Site 3 (N=4) | 1 | 1 | 2 | 0 | 1 | 1 | 0 | 0 |
| Site 9 (N=3) | 2 | 2 | 2 | 1 | 0 | 0 | 1 | 1 |
| Site 7 (N=3) | 2 | 2 | 2 | 1 | 1 | 1 | 1 | 1 |
| Site 1 (N=3) | 2 | 1 | 2 | 1 | 0 | 1 | 1 | 0 |
| Site 2 (N=3) | 2 | 2 | 2 | 1 | 2 | 1 | 0 | 0 |
| Site 4 (N=3) | **3** | **3** | **3** | 2 | **3** | **3** | **4** | 2 |
| Site 5 (N=3) | 2 | 2 | 2 | 1 | 1 | 2 | 2 | 1 |
| Site 11 (N=4) | 2 | 2 | 2 | 1 | 1 | 1 | 1 | 0 |
| Site 10 (N=4) | 2 | 2 | 2 | 1 | 2 | 1 | 1 | 1 |
| Site 8 (N=4) | 1 | 1 | 1 | 0 | 0 | 0 | 0 | 0 |
| Site 6 (N=4) | 2 | 2 | 2 | 2 | **3** | 1 | **3** | 2 |

***In institutions 3 and 7, two children were included in the classroom but excluded from the analysis because one parent of the paired controls rejected randomization to treatment as usual group; for the second, the site was unabale to find a paired control. Therefore the total N in the experimental group was finally 36 and not 38.**

**Criteria 1: number of sessions per week** (the protocol imposes four sessions per week for each child)

1: very few missed sessions

2: a few sessions missed due to the illness of a child or team members

3: one or two sessions were quite often missed

4: sessions were often missed

5: Many sessions were missed.

**Criteria 2: duration of the sessions** (the protocol requires two and a half hours or two hours the children are too young (<6 years))

1: two and a half hours almost constantly

2: two hours because of the difficulties for the institution to do more time

3: sometimes reduced to one hour and a half due to the constraints of the institution

4: the time of the sessions is often reduced (>2 times per week)

5: Many deviations regarding duration

**Criteria 3: The composition of the team is constant with one teacher from the school and the number of assistants needed for one-to-one ratio**

1 : very few absence of adults

2: a few adults are sometimes missing but an organization allows to work adequately with one adult and two children

3: a few short periods without the number of adults or the school teacher (<1 week)

4: relatively long periods with a deficient team or untrained people (<1 month)

5: long periods of protocol deviance (>3months)

**Criteria 4: Maintaining the number of school activities (n=6) and the number of groups (n=3)**

0: very few deviations

1: on short term, one month over two years (five instead of six activities) due to children's fatigue in adapting.

2: over a longer period, two months out of three years due to institutional difficulties

3: over a longer period, at least three to four months over three years due to institutional difficulties

**Criteria 5: Notes on children's behaviour and progress** (To be completed daily and weekly on the protocol grid**)**

0: no gaps or delays

1: a few gaps or a week late

2: the weekend meeting is sometimes not assured

3 : presence of delays in the delivery of notes and quotations as been frequent (>2 per months)

4 : at least one month delay in the delivery of the notes

5: delay in entering and returning notes by two to three months.

**Criteria 6: Local research coordinator involvement**

0: strong and constant involvement

1: a few moments of withdrawal from the commitment

2: irregular involvement

3: team reporting poor support from local coordinator

4: Local coordinator often delegated the function (>2 per months).

5: Local coordinator was not commited at all and mainly delegated the function.

**Criteria 7: Adaptation of the activities according to children’s developmental curriculum**

0: constant adaptation to the children developmental needs

1: difficulties in adapting to children needs at the beginning of the search

2: difficulties in identifying child-specific difficulties

3: difficulties for adults to find their place in the treatment model leading to constant deviations

4 : presence of children who are too difficult for the team

**Criterion 8: Ability to take into account remarks after supervision**

0: constant desire to learn and improve

1: minor difficulties to change after supervision

2: frequent difficulties to change after supervision

3: momentary obstacles due to rejection of a child

4: rejection of the way of implementing the protocol
